# Hepatocellular Proliferation Correlates with Inflammatory Cell and Cytokine Changes in a Murine Model of Nonalchoholic Fatty Liver Disease

**DOI:** 10.1371/journal.pone.0073054

**Published:** 2013-09-09

**Authors:** Michael N. VanSaun, Alisha M. Mendonsa, D. Lee Gorden

**Affiliations:** 1 Department of Cancer Biology, Vanderbilt University Medical Center, Nashville, Tennessee, United States of America; 2 Department of Surgery, Vanderbilt University Medical Center, Nashville, Tennessee, United States of America; University of Bari Medical School, Italy

## Abstract

Nonalchoholic fatty liver disease (NAFLD) is a problem of increasing prevalence and clinical significance worldwide and is associated with increased risk of development of end stage liver disease and cirrhosis, and can be complicated by hepatocellular carcinoma (HCC). NAFLD is characterized by physical and molecular changes in the liver microenvironment which include an influx of inflammatory cell populations, fibrosis, changes in gene expression, and cytokine production. To better understand changes to the liver in the setting of steatosis, we used a murine model of diet induced hepatic steatosis and corroborated our results with human patient samples of NAFLD. Among the cellular changes, we identified a significant increase in hepatocellular proliferation in the setting of steatosis as compared to controls. Analysis of inflammatory cell populations revealed increased infiltration of CD11b positive myeloid and CD3 positive lymphocytic cell populations in steatotic livers compared to normal livers. Resident Kupffer cells of the liver comprise the largest percentage of these myeloid cells and appear to be responsible for important cytokine alterations impacting proliferation of cells in the liver microenvironment. Significant alterations in cytokine profiles in the plasma and liver tissue lysates from normal and steatotic mice were detected including leptin, CXCL1, CXCL2, and CXCL16 that were further shown to directly increase hepatocyte proliferation *in vitro*. This increased hepatocellular proliferation and turnover in the setting of steatosis may play important roles in the progression and complications of NAFLD.

## Introduction

Nonalcoholic fatty liver disease (NAFLD) is commonly associated with obesity, the metabolic syndrome and type II diabetes mellitus and thus its significance parallels that of the epidemic rise of these diseases in this country and much of the world [1]. NAFLD can present as a spectrum of pathology ranging from benign steatosis, defined by triglycerides and other glycerophospholipids within hepatocytes of the liver and progress, to non-alcoholic steatohepatitis (NASH) characterized by the development of concomitant inflammation in the liver. Steatohepatitis is a unique liver microenvironment typified by accumulation of triglycerides, characteristic pathologic findings such as Mallory bodies as well as the infiltration of inflammatory cells as the disease progresses to steatohepatitis [2] [3]. Over time, this can progress to end-stage liver disease with fibrosis and cirrhosis. Some estimates suggest that NAFLD may be present in 17–33% of the U.S. population and that 33% of these patients have a significant component of NASH [4]. Currently 4–10% of liver transplants in the U.S. are performed for end stage liver disease due to NASH [5]. Equally ominous, is the increasing recognition that NASH and progressive liver fibrosis in this setting are risk factors for primary hepatocellular cancer.

Obesity is a recognized independent risk factor for the development of a number of epithelial malignancies including breast, colon and hepatocellular carcinoma (HCC) [6]. As many as 10% of patients with end stage liver disease due to NASH have concomitant hepatocellular carcinomas [6], [7]. In addition, there are increasing reports of HCC developing in the background of NASH, without accompanying cirrhosis [8]. Preclinical studies have shown that hepatic steatosis increases both development of primary hepatocellular cancer growth as well as the seeding of metastatic tumors [6], [9], [10]. In accordance with these reports, previous studies from our lab have shown that there is an increase in the number of metastatic tumor foci in the liver in the setting of steatosis using a mouse model of diet induced steatosis. Mice fed a high fat diet for over 9 months also develop spontaneous premalignant adenomatous tumors [10]. The importance of the steatotic change in the liver microenvironment for the establishment and growth of primary and metastatic tumors is not clearly defined. Obesity associated alterations in cytokine levels leading to increased levels of reactive oxygen species may evoke proliferative response from the hepatocytes [11]. There is mounting evidence that alterations in inflammatory mediators and cytokines as well as other factors such as insulin resistance, lipotoxicity and other metabolic regulators such as leptin, adiponectin and TNF-α have been implicated in the progression of NAFLD and liver fibrosis, and may also be important in tumorigenesis [12].

The liver is comprised of several resident cell types, which can contribute to recruitment of circulating inflammatory cells [13]. Hepatocytes comprise 60% to 80% of all liver cells and conduct the metabolic, biosynthetic, detoxification and biliary secretory functions of the liver. During development of steatosis, hepatocytes accumulate lipids and stain positive for triacylglycerides (TG). Accumulation of tryiglycerides in hepatocytes leads to generation of lipid metabolites such as lysophosphatidylcholine (LPC) and is associated with endoplasmic reticulum (ER) stress, c-Jun NH(2)-terminal kinase (JNK) activation that leads to lipoapoptosis of hepatocytes [14] [15]. Lipoapoptosis in turn leads to the recruitment of inflammatory cells contributing to the progression to NASH. Subsequently, NASH elicits pathological elements of hepatocellular injury, evident as cellular ballooning, appearance of Mallory bodies and apoptosis which exacerbates NAFLD.

As the epidemic of NAFLD increases, improved understanding of the changes in inflammatory cell populations and concomitant release/activation of cytokines in this unique liver microenvironment is necessary in order to develop strategies that could modulate these for therapeutic benefit. Importantly, studies of murine models that recapitulate human disease are crucial for translational studies to succeed. This study demonstrates significantly increased hepatocyte proliferation, alterations in serum and tissue cytokine levels, as well as local recruitment of inflammatory cell populations in livers in the setting of high fat diet induced steatosis.

## Materials and Methods

### Ethics Statement

All animal experimental procedures and protocols were approved by the Vanderbilt University Medical Center IACUC protocol #M/09/216 and performed according to institutional ethical guidelines for animal care and use. Human de-identified tissue samples were obtained from the Vanderbilt Translational Pathology Resource Core under CHTN 3 U01 CA091664-10S1. Human tissue samples were collected under National Cancer Institute (NCI) Best practices and CHTN standard operating procedures.

### Human Samples

Formalin-fixed, paraffin-embedded tissue samples and Optimal Cutting Temperature (OCT) embedded frozen tissue samples from normal and steatotic patients were obtained from the Vanderbilt Translational Pathology Shared Resource. Human liver samples were reviewed and diagnoses were assigned as either Normal (<5% Steatosis, n = 4) or Steatosis/Steatohepatitis (>20% Steatosis, n = 5) as determined by a Vanderbilt University pathologist.

### Mice

C57bl/6J male mice were obtained from Jackson Research Laboratories (Bar Harbor, ME) at 8 weeks of age and housed in an accredited laboratory animal facility at Vanderbilt University. On receipt, the mice were separated into appropriate cages and fed either a 13.5% fat “lean” diet (RD, 5001, LabDiet: 13.5% calories from fat, 58% from carbohydrates, and 28.5% from protein) or fed a 42% fat “high fat/western-style” diet (HF, TD.88137, Harlan Teklad (North America): 42% calories from fat, 42.7% from carbohydrates, and 15.2% from protein) *ad libitum* for 3 months.

### Tissue Samples

At the end of 3 months, mice were sacrificed and weighed. To isolate liver cells from both normal and steatotic mice, mice were anesthetized and an incision was made in the abdomen cavity. Hepatic tissue was dually perfused, first through the heart and then though the portal vein with heparinized Krebs Ringer Buffer (KRB) (154 mM NaCl, 5.6 mM KCl, 5.5 mM Glucose, 20.1 mM HEPES, 25 mM NaHCO_3_, pH7.4) to remove any intravascular blood cells. The liver was then perfused with 2.5 mls warm heparinized KRB (37°C) containing collagenase IV (500 U/ml), DNase I (1500 U/ml), CaCl_2_ (2.5 mM) and MgCl_2_ (2 mM) through the portal vein. Post perfusion, livers were removed and weighed. For flow cytometric analysis, 1 gram of liver tissue was transferred to a MACS C tube, processed on cycle A of the gentle MACS dissociator in 5 ml of the KRB collagenase solution. The resulting tissue suspension was incubated at 37°C for 30 min on a MACSmix tube rotator. Following enzymatic digestion, the C tube was put back on the MACS dissociator and processed with liver cycle 02. The suspension was passed through a 40mm filter and 20 ml of ice cold PEB (Phosphate buffered saline pH7.2, 2 mM EDTA, 0.5%BSA; Miltentyi Biotec) with DNase I (1500 U/ml) was added to the filtrate and centrifuged at 30 g for 6 minutes to pellet out the hepatocytes. The supernatant was collected and centrifuged at 300 g for 10 minutes to pellet the inflammatory cells, which were subsequently processed for flow cytometry. The remaining tissue samples of each liver were either fixed in buffered formalin, frozen in OCT compound, or homogenized in RIPA buffer (10 mM Tris pH 7.5, 150 mM NaCl, 0.1% SDS, 0.5% deoxycholate, 1% Triton) with addition of a complete Mini protease inhibitor cocktail tablet (Roche Diagnostics, Indianapolis, IN) for protein analysis. Additionally, endpoint heparinized blood samples were collected prior to perfusion and centrifuged at 2000×g for 20 minutes at 4oC to collect plasma for cytokine array analysis. All samples not immediately used were stored at −80°C.

### Histology

Formalin-fixed, paraffin-embedded tissue samples from both mouse and human samples were cut at 6 μm on a Leica microtome, dried, and then re-hydrated with xylenes and a decreasing ethanol series. For antigen retrieval, hydrated sections were boiled in a citric acid solution (10 mM trisodium salt dihydrate pH 6.0, 0.5% Tween-20) for 8 minutes. OCT embedded frozen tissue sections were cut at 8 μm on a Microm HM550 cryostat, air dried and fixed in ice cold acetone for 10 minutes. Slides were stained with antibodies directed against: Ki67 (Abcam, ab15580), HNF4α(Santa Crux, sc-655), CD68 (eBioscience, 12-0689-71), CD3 (BioLegend, 300415), CD8 (BioLegend, 301008) and CD56 (BioLegend, 318327). For diaminodenzidine, sections were labeled with appropriate species specific biotinylated secondary antibody (Vector Labs, Burlingane, CA), processed with a Vectastain kit (Vector Labs) and developed in chromogen solution (0.1 M Tris-HCl pH 7.4, 1.125 mM diaminobenzidine, 0.01% H_2_O_2_), counterstained with Mayer’s Hematoxylin Solution (Sigma), dehydrated with ethanols and mounted with permount. Slides were imaged with a Q Imaging Micropublisher color digital camera mounted to a Zeiss Axioplan 2 microscope using MetaMorph software for aquisition. For immunofluorescence, sections were labeled with anti rabbit Alexa Fluor (594) conjugated secondary antibody and counterstained with DAPI (4',6-diamidino-2-phenylindole, dihydrochloride). Fluorescent images were acquired with a Hamamatsu Orca ER CCD camera mounted to a Zeiss Axioplan 2 Microscope using MetaMorph software acquisition (Molecular Devices, Dowington, PA). The number of total cells, the number of hepatocytes and the number of proliferating cells were determined using nuclear markers by quantifying the number of DAPI positive, HNFα positive and Ki67 positive nuclei respectively. This was done by thresholding images to a preset background level through imageJ software and measuring the total number of particles over 75 pixels, to eliminate background and select only nuclei. Average number of cells from five random 20x fields per human (n = 4) and mouse (n = 5) sample were obtained. Inflammatory cell quantification of immunohistochemical (IHC) staining in human samples was carried out by setting a threshold to a fixed intensity and calculating the percent thresholded area with metamorph software analysis. For IHC analysis, the average thresholded area from five random 10x fields per human sample (n = 4) were obtained. Statistical analysis was carried out using *t*-test with GraphPad Prism software.

### Flow Cytometry

Pelleted inflammatory cells from digested livers were resuspended in PEB. CD45 or CD11b positive cells were isolated using positive selection with magnetic beads. 20 μl of respective magnetic beads (Miltenyi Biotec) were incubated with the pelleted cells for 20 minutes on ice, inverting the tubes every 5 minutes. Cells were spun down at 300 g, resuspended in PEB, filtered through 0.3 um mesh and magnetically isolated via LS columns (Miltenyi Biotec) according to manufacturer’s instructions. Total number of cells, isolated by CD45 magnetic beads, was determined per gram of liver tissue with a BioRad TC10 automated cell counter. CD45 isolated cells were resuspended in 250 ul of staining solution containing lineage specific markers. Markers used for mouse T-cell subpopulations staining were CD3 (488), CD4 (A700), CD8 (PE), CD25 (APC) and CD62L (e-Fluor 450). Granulocytes were stained with F4/80 (APC), GR1 (PE), Ly6C (e-Fluor 450). Dendritic cells were identified with monoclonal antibodies to CD11c (e-Flour 450) and B-cells were revealed as CD19 (APC) positive. 7-AAD was used as a vital stain. All fluorophore-conjugated monoclonal antibodies were obtained from BD Pharmingen. Cells were labeled for 20 minutes at room temperature with constant agitation on a Nutator. Post staining, cells were washed twice with 1ml PEB and resuspended in a final volume of 250 μl. Flow cytometry data acquisition was performed on a 3-laser BD LSRII at the VMC Flow Cytometry Shared Resource. Five mice per group were used for flow cytometric analysis. FlowJo software was used for data and statistical analysis of flow cytometric results. Additionally, magnetically isolated CD11b positive cells were spun at 300 g and resuspended in RPMI media and 150,000 cells per well were cultured for 24 hours before isolating conditioned media for cytokine analysis. Statistical analysis was compared using GraphPad Prism software and compared using two-tailed unpaired T-test.

### Cytokine arrays

Liver tissue lysates from normal and steatotic mice were quantified using the BCA assay (Pierce). 100 micrograms of protein lysate was added to each array (n = 3). Plasma collected from both normal and steatotic mice was diluted 1:10 in blocking buffer and 100 μl of sample was used for cytokine array analysis (n = 3). Additionally, 100 μl of pooled conditioned media collected from CD11b positive cells isolated from both normal (n = 3) and steatotic mice (n = 3) was run for cytokine profile analysis. Cytokine array analysis was carried out using RayBiotech Mouse cytokine arrays (AAM-CYT-G3) as directed by their protocol. Briefly, the array surface was first blocked and then incubated with sample overnight. Arrays were washed and subsequently incubated with fluorescently tagged secondary antibody. Stained slides were scanned using a GenePix 4000B Microarray Scanner at the Vanderbilt VANTAGE Core. Densitometric analysis was then performed using GenePix Pro Acquisition and Analysis Software. Background was subtracted and data were normalized against positive controls included on each array. All data directly compared were derived from the same batch of arrays. Statistical analysis for fold change was performed with Microsoft Office Excel.

### Cell lines

HepG2 human hepatocellular carcinoma cells were obtained from American Tissue Culture Collection (ATCC, Rockville, MD, USA) and maintained as recommended in Dulbecco’s modified Eagle’s medium (DMEM; Life Technologies) containing 10% fetal calf serum. HEPT mouse hepatocyte cells were isolated from the immortomouse expressing the temperature sensitive SV40 gene encoding large T antigen [16]. The HEPT cells are grown in DMEM with 10% FBS, P/S, L-glutamine and ITS (insulin, transferrin, selenium, Gibco 41400045), 1U/ml IFN-γ and incubated at 32°C in 5% CO_2_. Prior to experimental analysis, cells were split and transferred to 37°C in 5% CO_2_ to deactivate SV40 for a minimum of 24 hours.

### Effect of cytokines on hepatocyte proliferation in vitro

The effect of Leptin, CXCL1, CXCL2 and CXCL16 cytokines on the growth of HepG2 human hepatocellular carcinoma cells and mouse SV40 transformed hepatocyte (HEPT) cells was determined using the MTT assay. In brief, 10,000 cells were plated per well in complete media in a 96 well plate overnight and allowed to attach to the plate overnight. Next morning, cells were serum starved in serum free media. After 24 hours of serum starvation, different concentrations of Leptin, CXCL1, CXCL2 and CXCL16 (100 ng/ml, 25 ng/ml and 1 ng/ml) were added to each well. Serum free conditions and 10% FBS were used as controls. After 12 hours, 20 μl of MTT reagent (5 mg/ml, Sigma) was added to each well and returned to the incubator for 2 hours after which the media was aspirated from each well and the remaining MTT formazan crystals dissolved in 100 μl of isopropanol. Absorbance at 570 nm was read using a Victor3 V 1420 Multilabel Plate Counter. Experiments were carried out in triplicate and statistical analysis was carried out with GraphPad Prism software applying a two-tailed unpaired T-test.

## Results

### Hepatocellular proliferation in steatotic livers

**Figure 1:**
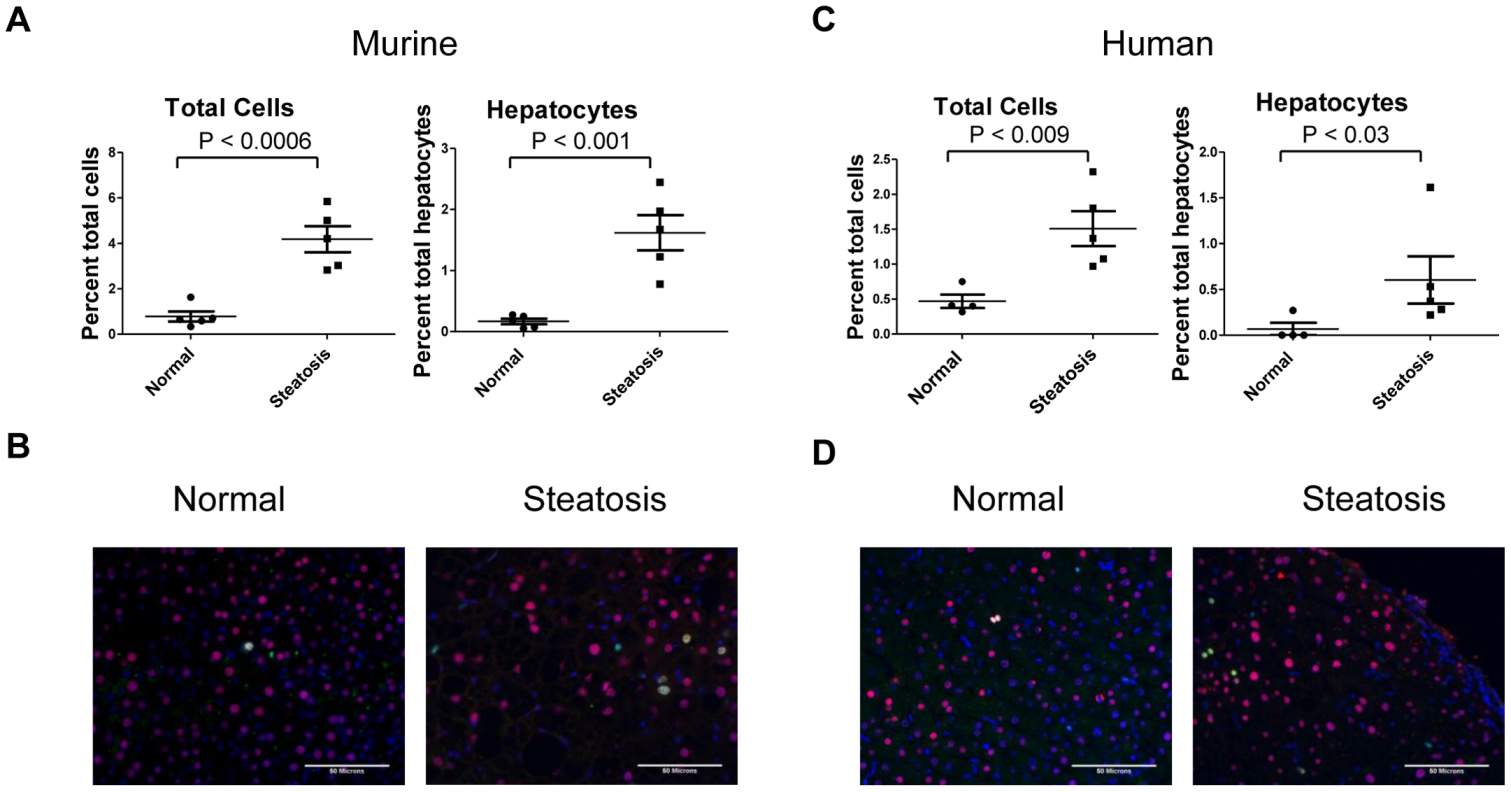
Steatosis results in increased cellular proliferation in the liver. The percentage of total Ki67 positive cells and the percent of Ki67/HNF4α double positive hepatocytes are both increased in steatotic livers compared to normal livers of (A, B) murine and (C, D) human samples. Representative images of Ki67 (green), HNF4α (red) and DAPI (blue) immunofluoresence staining in livers of normal and steatotic (B) murine and (D) human liver sections showing increased number of Ki67 positive cells in steatotic livers. Images are taken at 40X and scale bars represent 50 microns.

To study the progression of NAFLD, we have used a previously validated mouse model of diet induced steatosis [10]. In this study, prolonged 42% high fat diet led to fibrosis, inflammation and development of dysplastic lesions in the liver. We therefore wanted to determine whether high fat diet induced hepatic steatosis led to changes in proliferation of cell populations within the steatotic liver. To do so, human and murine liver tissues with and without steatosis were triple stained for the nuclear markers Ki67, HNF4α and DAPI. HNF4α has previously been demonstrated to specifically stain hepatpatocytes in the liver [17]. The overall number of proliferating Ki67 positive cells as a percentage of total number of DAPI positive cells as well as the percentage of co-positive Ki67/HNFα proliferating hepatocytes vs total HNF4a positive hepatocytes were counted and quantified per field. We found a significant increase in the percentage of total Ki67 positive cells as well as the percentage of Ki67 positive hepatocytes in both human and murine steatotic liver samples vs normal liver samples (Figure 1). These results indicate that as NAFLD correlates with an increase in the number of proliferating hepatocytes in the liver.

### Inflammatory cell population changes in the murine steatotic liver

**Figure 2:**
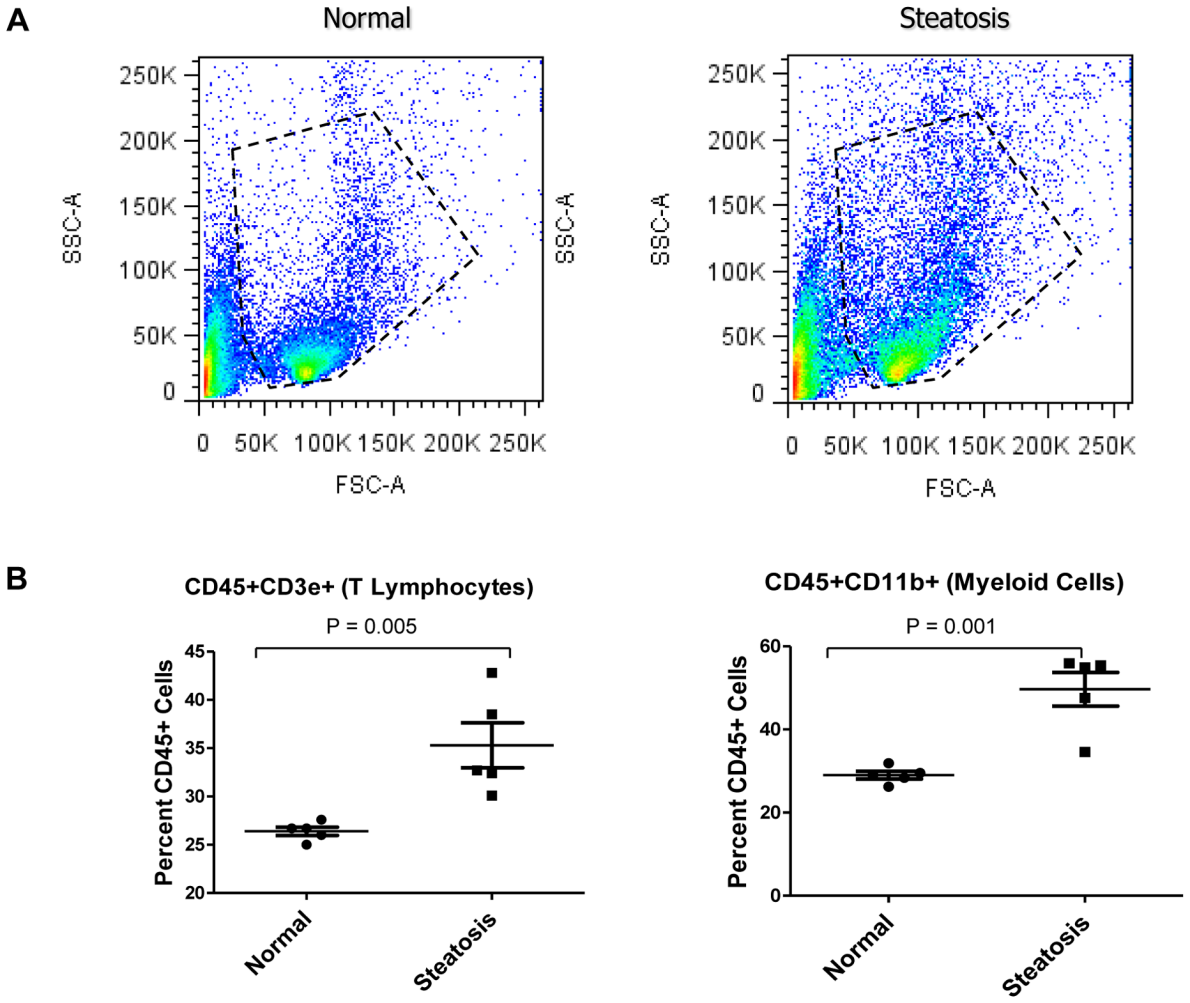
Steatosis results in changes in the inflammatory cell populations in the murine liver. CD45 positive cells were isolated from normal and steatotic digested liver samples by immunomagnetic beads and then stained for the immune markers CD3 and CD11b before being subjected to Flow cytometric analysis. (A) Diagrams are representative scatter plot of flow cytometric analysis for overall CD45 positive cells in the livers. (B) CD45 positive cells were then gated for the percentage of CD3 positive T lymphocytes or CD11b positive myeloid cells. CD3+ and CD11b+ subpopulations were both increased in the steatotic livers versus the control livers.

**Figure 3:**
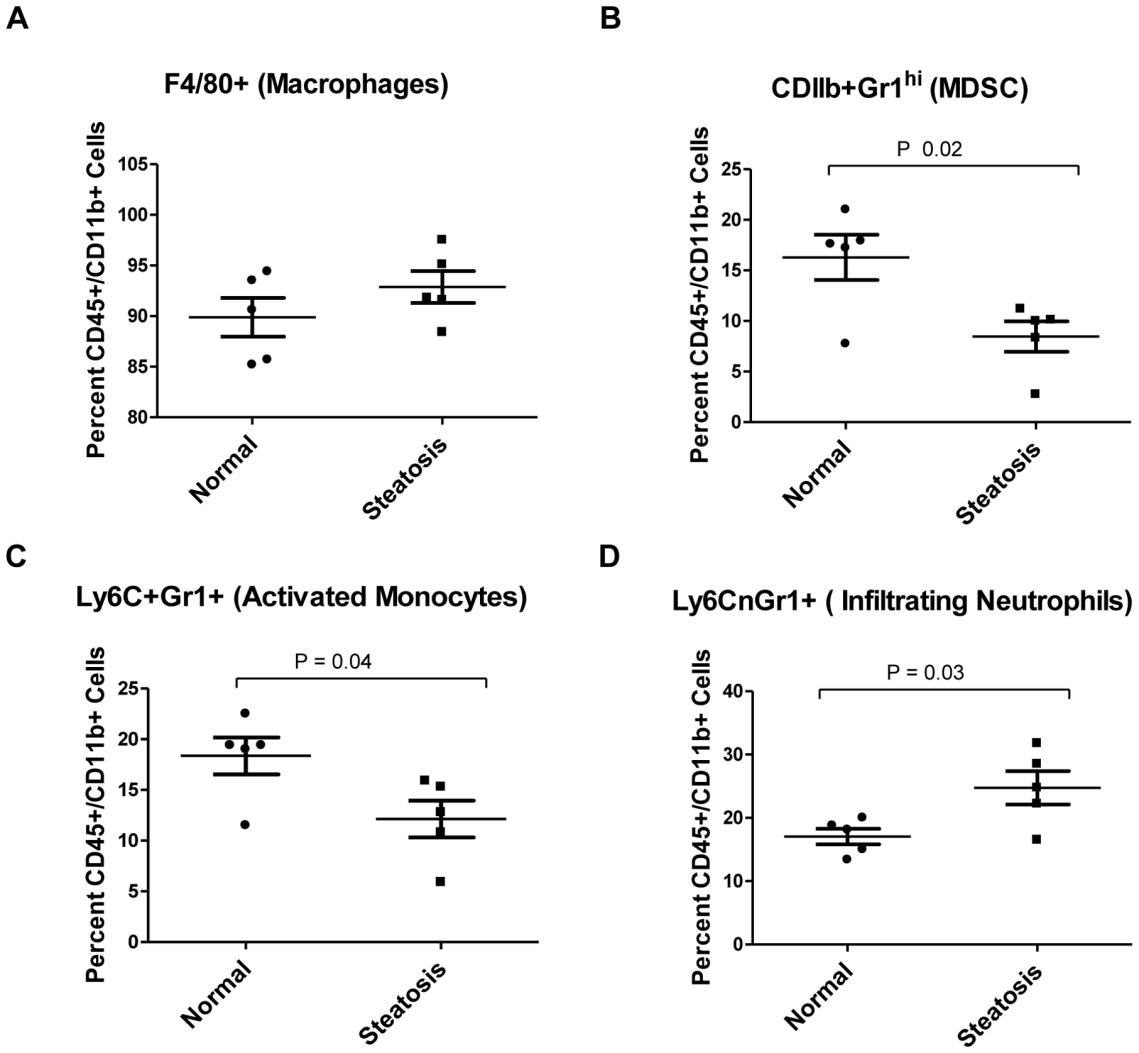
Changes in the myeloid cell sub-populations in normal vs steatotic murine livers. Isolated CD45 positive cells from normal and steatotic livers were stained for myeloid markers, subjected to flow cytometry and gated as a fraction of CD11b positive myeloid cells. (A) CD11b+ cells were analyzed for changes in the total percentage of F4/80 positive macrophages, (B) Gr1^hi^ expressing cells, (C) Ly6C positive Gr1 positive activated monocytes and (D) Ly6C negative Gr1 positive infiltrating neutrophils between normal and steatotic murine livers. Results show a significant decrease in the GR1hi and Ly6CpGr1p subpopulations and a significant increase in the Ly6CnGr1p subpopulation of steatotic versus normal livers, while there was not a significant difference the percentage of F4/80p subpopulation.

As NAFLD progresses to NASH, it is characterized by an influx of circulating inflammatory cells and an alteration in the subpopulations of local inflammatory cells. Studies have shown a significant increase in CD45 positive hematopoietic inflammatory cells in mice with diet induced steatosis [18]. To profile early changes in inflammatory infiltrates in the steatotic liver, samples were characterized for the effect of high fat diet induced steatosis on various inflammatory cell populations by flow cytometry analysis. CD45 positive inflammatory cells were isolated from dissociated livers using magnetic beads and a positive selection. Absolute number of CD45 positive cells per gram of liver tissue were counted and demonstrated a significant increase in the steatotic livers versus normal livers (8.027×10^6^±0.38×10^6^ and 4.84×10^6^±0.32×10^6^ respectively, p<0.0029). Changes in inflammatory cell populations were further represented as a percentage of the isolated CD45 positive cells. High fat diet induced steatosis resulted in an overall increase in the CD45/CD11b positive myeloid cell population and the CD45/CD3e positive lymphocytic population (Figure 2B). Next, to look at whether there were changes in the individual myeloid cell subpopulations, cells were co-stained with F4/80, Gr1 and Ly6C to look at differences in macrophage, activated monocytes, infiltrating neutrophil and MDSC cell populations after gating for CD11b as shown in Figure 3. No significant difference was observed in the percentage of the subset of F4/80 positive macrophages, however there was a significant decrease in the percentage of GR1^hi^ expressing MDSC’s and Ly6C^+^Gr1^+^ activated monocytes while there was a decrease in the percentage of Ly6C^n^Gr1^+^ infiltrating neutrophils in the steatotic livers compared to the normal livers.

**Figure 4:**
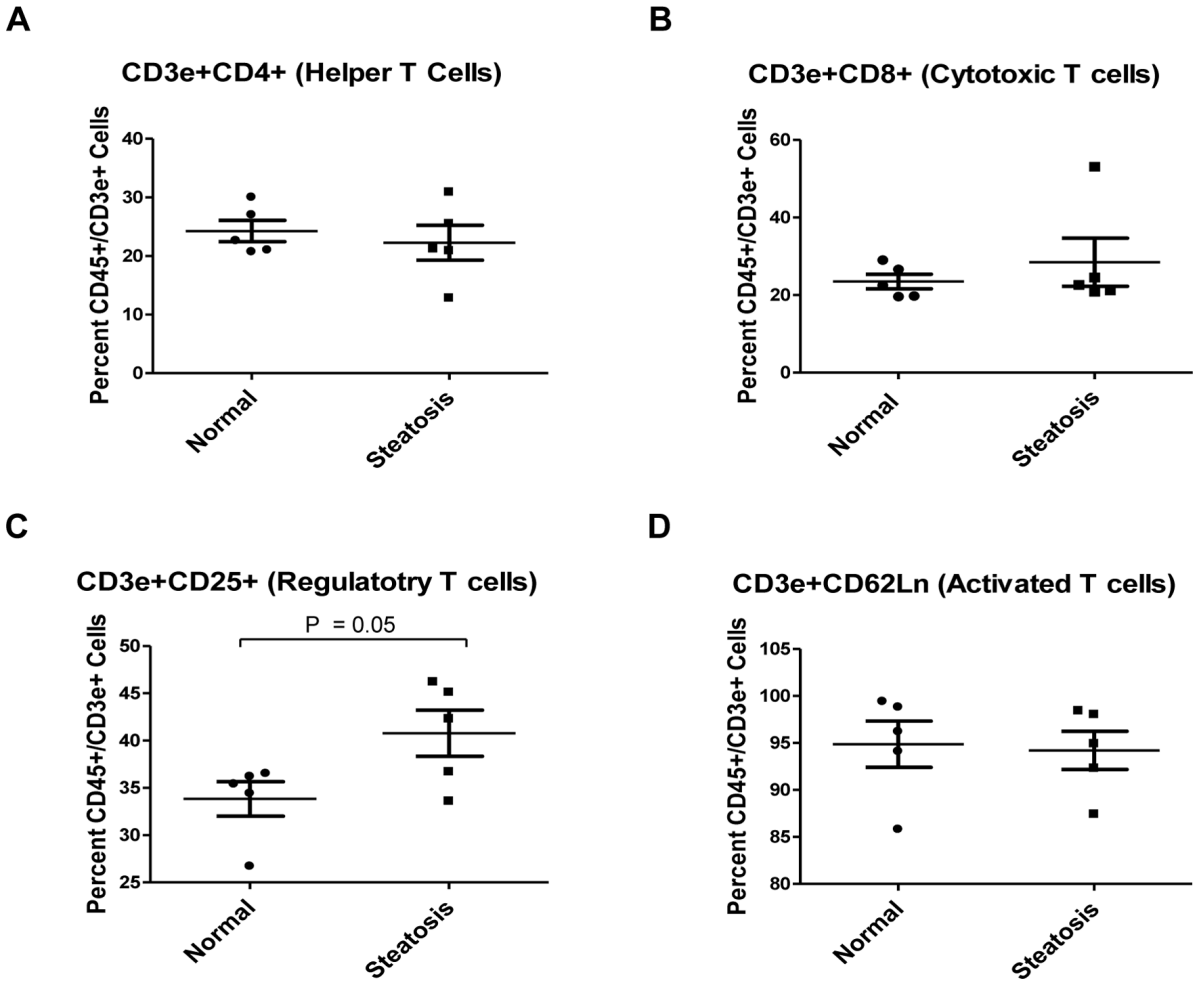
Changes in the lymphoid cell sub-populations in normal vs steatotic murine livers. Isolated CD45 positive cells from normal and steatotic livers were stained for lymphoid markers, subjected to flow cytometry and gated as a fraction of CD3e positive lymphocytic cells. CD3e^+^ cells were analyzed for changes in the total percentage of (A) CD4 positive helper T cells, (B) CD8 positive cytotoxic T cells, (C) CD25 positive regulatory T cells, and (D) CD62L negative activated T cells between normal and steatotic murine livers. Results only showed a significant increase in the overall CD25^+^ subpopulation of CD3e^+^ cells in the steatotic livers when compared to normal livers.

The lymphocytic cell population was identified from CD45 positive isolated cells by staining for CD3e and additionally T cell subpopulations were identified with markers for CD4, CD8, CD25 and CD62L (Figure 4). From the overall increase in CD3e^+^ cells in steatotic livers, we did not detect any significant differences in percentage of T cell subpopulations except for a slight increase in the total number of CD25 positive regulatory T cells in steatotic livers compared to normal livers (p = 0.05).

**Figure 5:**
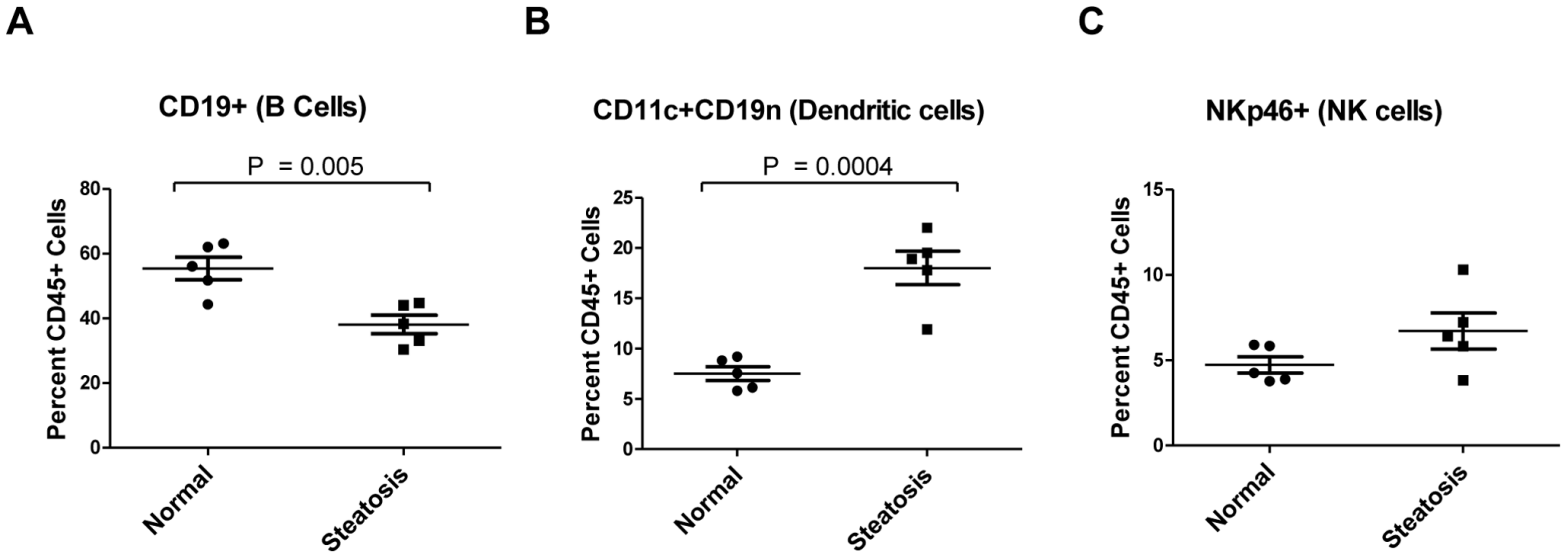
Changes in cell sub-populations of B cells, Dendritic cells and Natural Killer cells in steatotic livers. Isolated CD45 positive cells from normal and steatotic livers were stained for markers for (A) B cells (CD19^+^), (B) dendritic cells (CD11c^+^, CD19^–^) and (C) natural killers cells (NKp46^+^) before being subjected to flow cytometry and gated as a fraction of CD45^+^ cells to detect total percentage of subpopulations. Results demonstrate a significant decrease in B cells and a significant increase in total dendritic subpopulations in steatotic murine livers compared to normal livers. Percentage of the overall subpopulation of natural killer cells were not significantly different between groups.

Additionally, CD45 positive cells were stained with CD19, CD11c and NKp46 to look at changes in B cell, dendritic cell and natural killer cell subpopulations respectively between the livers of normal and steatotic mice (Figure 5). There was a significant decrease in B cell subpopulation, an increase in dendritic cell population and no difference in the natural killer cell subpopulation in the steatotic livers. To ensure inflammatory changes were specific to the steatotic liver and not generally associated with effects of obesity in other organs, we additionally profiled inflammatory subpopulations from the spleen. The resident inflammatory cell subpopulations in the spleen are shown in the supplementary data (Table S1).

### Inflammatory cell population changes in the setting of human NAFLD

**Figure 6:**
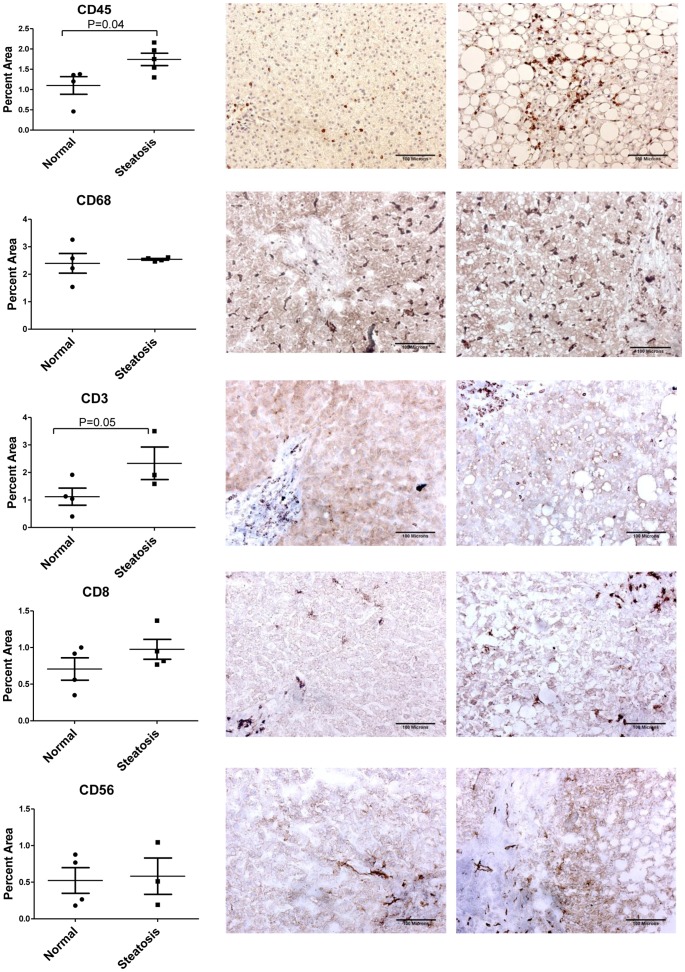
Quantification of inflammatory cell populations in human liver samples. Immunohistochemical staining was used to detect and quantify changes in inflammatory cell populations between normal and steatotic human liver samples. Positive immunoreactive staining (dark brown) was calculated as a percentage of total area for (A) CD45 positive inflammatory cells, (B) CD68 positive macrophages (C) CD3 positive T lymphocytes (D) CD8 positive cytotoxic T cells and (E) CD56 positive NK cells in frozen sections of normal and steatotic human livers. Results demonstrate a significant increase in the number of CD45 and CD3 positive cells in the steatotic livers when compared to normal liver samples. CD68 macrophages, CD8 cytotoxic T cells, and CD56 NK cells were not significantly altered between samples. Images are 20x.

In order to test whether changes seen in inflammatory cell populations using the mouse model of high fat diet induced steatosis were corroborated in human samples with NAFLD, frozen liver sections were obtained from patients with and without steatosis and stained for inflammatory cell markers CD45, CD68, CD3e, CD8 and CD56 as shown in Figure 6. We detected a significant increase in overall CD45 positive cells (P = 0.04) as well as the total T lymphocytes as determined by the positive staining for CD3e marker (P = 0.05) in steatotic livers. Staining for the CD8 subpopulation of T cells showed a slight increase in the steatotic livers, although this increase was not statistically significant. No significant differences were observed in the percent area stained for macrophages (CD68) or NK cells (CD56) by immunohistochemical analysis of normal and steatotic human liver samples, consistent with our findings in the steatotic murine livers.

### Changes in cytokine profiles in a murine model of NAFLD

Changes in inflammatory cell populations are associated with activation and secretion of various cytokines such as TNFα, IL-6, MCP-1, IL-10, have been shown to be elevated in patient serum samples in the setting of NASH [9], [19]–[21]. These specific cytokines have additionally been proposed to play critical role in NAFLD pathogenesis [22]–[25]. To examine changes in additional cytokines we utilized a commercially available cytokine array to quantify the relative changes in various cytokine levels circulating in the plasma, in the liver tissue proper, and cytokines secreted from isolated CD11b positive myeloid cells of normal versus high fat diet induced steatotic mice. The cytokine array also contained some additional relevant adhesion molecules associated with the array. Array proteins that were significantly different are reported in Table 1 for each respective group. When comparing arrays from steatotic vs normal mice, several cytokines and relevant adhesion molecules were increased in both plasma and liver lysates, including Axl, CXCL16, Eotaxin, IL-13, IL-2, Leptin, CXCL4 (Platelet factor 4), P-selectin and VCAM-1. Further, CTACK (Cutaneous T cell-attracting chemokine), IL-6, IL-3 Rb and SCF (Stem cell factor) were elevated in the liver lysates and not plasma of steatotic mice. CTACK and IL-9 were significantly reduced in the plasma of steatotic mice when compared with plasma from normal mice.

**Table 1:** Fold change in cytokine levels of high fat diet fed mice.

Cytokine	Plasma	Liver Lysate	CD11b^+^ Cell CM
Axl	2.13280389	1.831922538	2.443505541
CTACK	0.21229807	2.179987169	0.75720946
CXCL16	1.93180689	1.58065464	4.028336666
Eotaxin	1.65832784	2.007739833	0.130455649
IL-13	1.36055055	1.768719618	0.473546075
IL-2	2.34419611	2.618608513	0.859891851
IL-3	1.1356281	1.519534855	0.651248631
IL-3 Rb	1.09612642	2.513275758	0.858366742
IL-6	1.06119747	1.40169613	0.864977387
IL-9	0.46101177	1.508281212	0.874990301
KC	1.72431495	1.406850455	4.207922861
Leptin	1.86116246	1.840166242	0.840621661
L-selectin	1.36488563	1.520783331	0.722070716
MCP-1	1.01054898	1.340761806	0.694198945
MCP-5	1.02686785	1.319179552	0.657637641
MIP-1γ	1.68656519	1.178307719	11.43207136
MIP-2	1.47759187	1.656782016	4.711865379
PF-4	1.73472362	3.080963553	0.983425609
P-selectin	1.73297843	2.002406638	0.983425609
RANTES	1.02225724	1.36710967	6.589157964
SCF	0.9931903	2.093534246	2.293077231
sTNF R1	0.72735291	0.994273435	1.778782317
sTNF RII	2.5148749	1.022674689	4.160571141
TNF-α	1.20435513	1.290203739	0.793791961
VCAM-1	1.91774475	1.574854392	0.886619198

Plasma, liver tissue lysates and conditioned media from CD11b^+^ magnetically isolated cells Cytokine arrays were used to detect changes in cytokine levels in the plasma, perfused liver tissue lysate (Liver Lysate), and from conditioned media of isolated CD45^+^CD11b^+^ subpopulations (CD11b^+^ Cell CM) between normal and steatotic samples. Cytokine values are presented as fold change with values greater than 1 representing increased levels and values less than 1 representing decreased levels in steatotic samples. (n = 3). Axl (Tyrosine protein kinase 7), CTACK (Cutaneous T-cell attracting chemokine, CCL27), CXCL16 (CXC chemokine ligand 16), IL-2 (Interleukin-2), IL-3 (Interleukin-3), IL-6 (Interleukin-6), IL-9 (Interleukin-9), IL-13 (Interleukin-13), IL-3 Rb (Interleukin-3 receptor beta, CD131), KC (CXC chemokine ligand 1), MCP-1 (Monocyte chemotactic protein-1, CCL2), MCP-5 (Monocyte chemotactic protein-5, CCL12), MIP-1γ (Macrophage inflammatory protein-1 gamma), MIP-2 (macrophage inflammatory protein -2, CXCL2), PF-4 (Platelet factor 4, CXCL4), RANTES (Regulated on activation normal T-cell expressed and secreted, CCL5), SCF (Stem cell factor), TNF (Tumor necrosis factor), VCAM-1 (Vascular cell adhesion molecule 1, CD106).

Kupffer cells have been shown to undergo activation and play a role in progression of various liver diseases by secretion of cytokines that lead to activation of stellate cells and chemoattraction of inflammatory cell populations to the liver [13], [26]. Therefore, CD11b cells were isolated from dissociated normal and steatotic livers and used to generate conditioned media. Kupffer cells represent the majority (85% to 95%) of the CD11b positive population in the liver. Cytokine arrays were used to analyze differences in secreted cytokines from conditioned media of CD11b positive cells isolated from normal and steatotic livers. MIP-1r, MIP-2, RANTES, KC, sTNF RII and CXCL16 were elevated in the conditioned media from CD11b positive cells isolated from steatotic versus normal livers (Table 1).

### Cytokines increase hepatocyte proliferation in vitro

**Figure 7:**
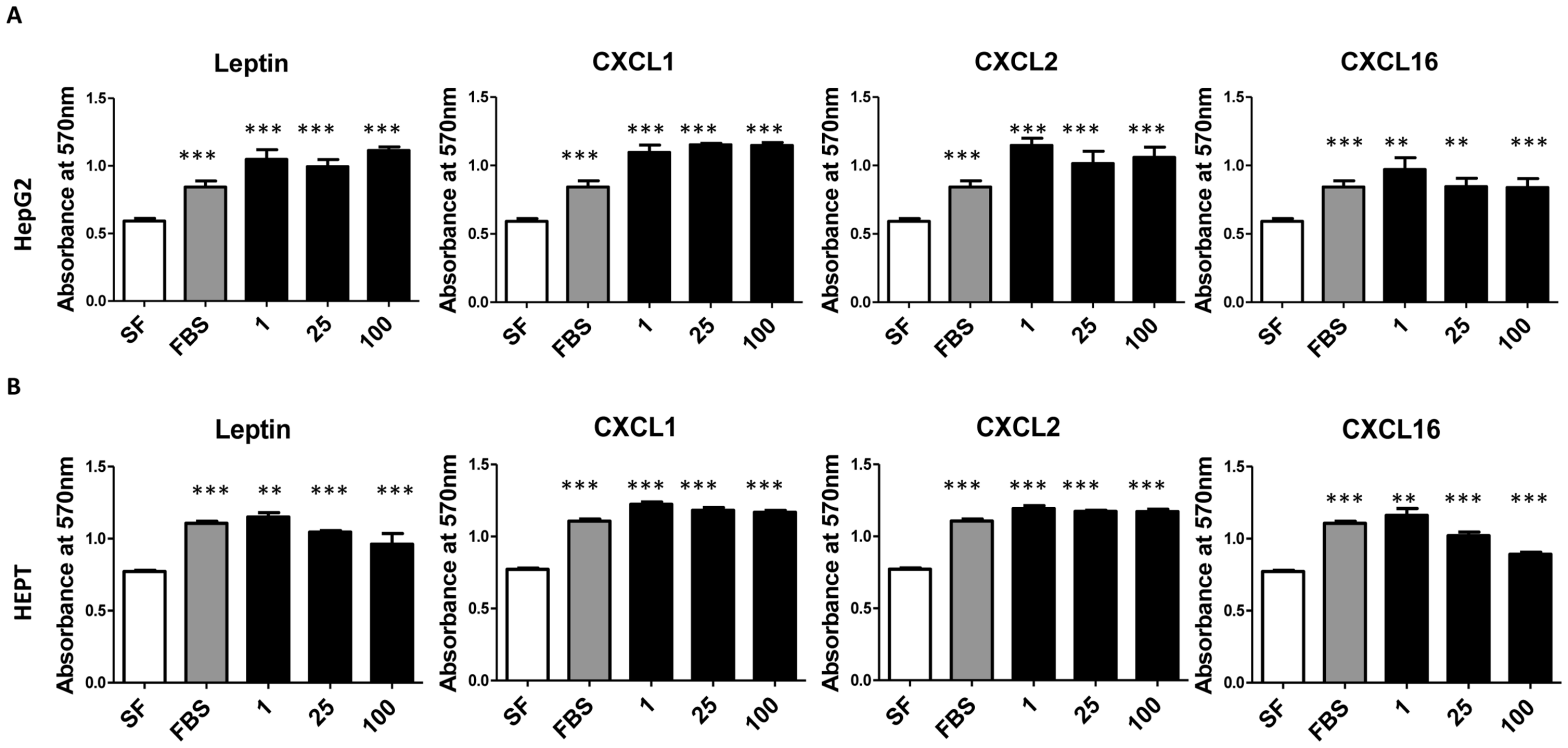
Cytokines effect hepatocyte proliferation in vitro. The effect of different concentrations of Leptin, CXCL1, CXCL2 and CXCL16 (100 ng/ml, 25 ng/ml and 1 ng/ml) on proliferation of (A) HepG2 and (B) HEPT cells determined by MTT assay. 10% FBS was used as a positive control. ** represents P<0.001 and *** P<0.0001 when compared to Serum Free conditions.

To determine whether alterations in cytokines could reflect changes in the observed increase in hepatocellular proliferation in the steatotic livers, we tested the effect of select cytokines (leptin, CXCL1, CXCL2 and CXCL16) that were increased in steatotic samples on hepatocyte proliferation. The effect of various cytokines on the growth of HepG2 human hepatocellular carcinoma cells and mouse SV40 transformed hepatocyte (HEPT) cells was determined using the MTT assay. Leptin, CXCL1, CXCL2 and CXCL16 significantly increased proliferation off both cell lines after 12 hours of treatment with different concentrations of each cytokine (Figure 7). Thus the increases in cytokine levels observed in the setting of steatosis could explain the increased hepatocellular proliferation determined by Ki67 staining.

## Discussion

With the rising incidence of obesity, NAFLD is an increasing cause of chronic liver disease in the United States and the world, encompassing a spectrum of pathology marked by hepatic steatosis in the absence of significant alcohol consumption. Although simple steatosis follows a generally benign course, the more aggressive form, non-alcoholic steatohepatitis, can progress to cirrhosis and result in complications including hepatocellular carcinoma. A significant number of cases of hepatocellular carcinoma are occurring in the setting of NASH without underlying cirrhosis [27], [28]. A number of cellular and molecular mediators have been shown to be involved in the progression of NAFLD and some of these may be linked to tumor initiation and progression in the hepatic microenvironment of NAFLD. Accumulation of lipids in the liver cells can lead to hepatocellular injury [3], [14], [15], one manifestation of which is apoptosis which in turn can trigger a regenerative response. Previous studies in our lab have shown that mice fed a high fat diet for a prolonged period of time develop liver tumors [10]. To determine whether steatosis, progressive inflammation, and hepatocellular injury could impact hepatocyte proliferation, the total number of Ki67 positive cells as well as Ki67 positive hepatocytes were quantified and showed a statistically significant increase in hepatocellular proliferation in the steatotic livers as compared to normal livers. Repetitive cycles of apoptosis and regeneration/proliferation of these principal cells of the liver cells could lead to aberrant repair in some individuals culminating in tumor initiation.

Our current studies, using a mouse model of diet induced steatosis have shown that there are significant changes in specific inflammatory cell populations in the liver in the setting of steatosis. We observed significant increases in myeloid cell, T and B lymphocytes and dendritic cells populations. Accumulation of tryiglycerides in hepatocytes leads to the generation of lipid metabolites such as lysophosphatidylcholine (LPC), which has been associated with oxidative stress and hepatocellular death [11], [14], [19]. Cell death can lead to activation of inflammatory pathways such as the JNK and NF-κB pathways through release of damage-associated molecular patterns (DAMPs), which can then lead to the recruitment of inflammatory cells and contribute to the progression to NASH[1], [13]–[15], [29]. Kupffer cells are resident macrophages and act as the first responders to hepatic injury, they likely detect the expression of DAMPs on hepatocytes which have been injured by accumulation of triglycerides in the setting of steatosis [30], [31]. The subsequent production of TNFα and other chemoattractant cytokines by Kupffer cells thus propagates the initial insult, leading to inflammation through the recruitment of inflammatory monocytes [32].

Recent studies by other groups have additionally shown that changes in kupffer cell and dendritic cell populations can play an important role in the progression of NAFLD. Work by Henning et.al. demonstrated an increase in CD11c^+^ dendritic cell populations and demonstrated a regulatory role for dendritic cells (DCs) in NASH by limiting sterile inflammation via their role in clearance of apoptotic cells and necrotic debris. They found that DCs increase regulatory T cell activation and production of the anti-inflammatory cytokine IL-10. Further, they showed that ablation of dendritic cells led to increased Toll-like receptor expression and cytokine production in innate immune effector cells in NASH, including Kupffer cells, neutrophils, and inflammatory monocytes [18]. In concordance with their data, we detected a significant increase in the percentage of CD11c^+^ dendritic cells in the high fat diet induced steatotic livers compared to normal livers. Though we were not able to detect any changes in the percentage of CD11b^+^F480^+^ cells (Kupffer cells) as a percentage of the CD11b^+^ myeloid cells between normal and steatotic livers, we did see a significant increase in the percentage of CD11b^+^ cells in the steatotic livers compared to normal livers. Additionally, we were able to detect changes in cytokine production from conditioned media (CM) obtained in vitro from isolated CD11b^+^ myeloid cells. Since the Kupffer cells make up the majority of this population (85%–95%), cytokine alterations reflect changes in the resident Kupffer cells of the liver and their activation status.

Multiple chemokines and cytokines have been implicated in the development of steatosis and the progression to NASH; including IL-6, TNF-α, MCP-1 and IL-10 [23], [24]. The development of NASH in human patients and in murine models of NASH have each exhibited elevated serum levels of TNFα and increased expression of TNF transcripts in liver as well as adipose tissue [9], [33], [34]. Our results comparing plasma and liver tissue samples from normal and steatotic livers, showed a slight increase in IL-6 and TNFα(Table 1). Park et al recently reported that proinflammatory cytokines IL-6 and TNFα are important for the progression from hepatic steatosis to steatohepatitis in obese mice and that absence of either IL-6 or TNFR1 reduced lipid accumulation in the liver and also reduced influx of macrophages and neutrophils in livers of mice fed a high fat diet [9]. However, other studies have shown that IL-6 deficiency or blockade reduced liver inflammation without affecting the development of steatosis suggesting a role for IL-6 only in promoting liver inflammation [35], [36].

MIP-1γ, MIP-2, RANTES, CXCL1, sTNFRII and CXCL16 represented the most significantly elevated cytokines specifically from the CD11b conditioned media. MIP-1γ has been shown to attract dendritic cells and immature myeloid cells that possess the CCR1 chemokine receptor [37] and its expression in tumor cells aids colon cancer metastasis to the liver and accumulation of immature myeloid cells [38]. Increased expression of MIP-2 has been shown to contribute to neutrophil and lymphocyte recruitment [39] which could help propagate the inflammatory response observed in NAFLD. CXCL1 is expressed by macrophages, neutrophils and epithelial cells, and has neutrophil chemoattractant activity. It is involved in inflammation and gene expression levels are elevated in patients with NASH [23], [40]. Recent studies have shown that elevated RANTES and sTNFRII levels correlate with the progression of NAFLD [41], [42] though further functional studies will need to be carried out for these and other altered cytokines in the setting of NAFLD. A recent study identified CXCL16 in preoperative serum as a marker for poor prognosis and high level of recurrence of liver metastasis in patients with HCC [43]–[45].

Specifically decreased in the conditioned media of CD11b cells from steatotic livers were IL-13, MCP-5 and MCP-1. IL-13 is a Th2 cytokine that plays a central role in various inflammatory diseases [46]. IL-13 induces tissue fibrosis by stimulating and activating TGF-β1 and was shown to play a role in progression from NASH to fibrosis in a rat model fed a choline deficient diet[47]. However, no studies have evaluated levels at early stages of NAFLD. MCP-5 specifically attracts eosinophils, monocytes and lymphocytes and is therefore found predominately in lymph nodes and thymus under normal conditions, yet its expression can be induced in macrophages [48]. It has been shown to play a role in exacerbation of pulmonary fibrosis by recruitment of bone marrow derived fibrocytes to the lung [49], [50], but its role in progression of NAFLD is yet to be assessed. Several studies have reported an important pathological role of MCP-1 in the progression of NAFLD. However we detected no significant differences in cytokine levels from plasma or tissue samples. In NASH, the role of MCP-1 is controversial; MCP-1 deficiency in mice fed a methionine choline deficient diet didn’t affect the development of steatohepatitis, but actually decreased fibrosis [21], and didn’t impact liver disease progression [51]. Recently, pharmacological inhibition of MCP-1 or the lack of CCR2 expression (MCP-1 receptor) in a murine model of NASH was shown to decrease liver inflammation and steatosis without affecting hepatic fibrogenesis [52], [53]. The current study provides evidence that various cytokines are differentially expressed during the early stages of NAFLD in a mouse model of diet induced steatosis and further studies are required to determine the precise role of each of cytokines at different stages of NAFLD.

While effects of microenvironmental as well as systemic alterations of chemokine levels have been demonstrated for the recruitment of inflammatory cells, less is known about the effects of these chemokines on hepatocytes. We were able to show that high fat diet induced steatosis results in a significant increase in hepatocyte proliferation compared to normal livers. Interestingly, the Canbay group was able to also demonstrate a significant increase in proliferation in steatotic livers after partial hepatectomy, which correlated with increased levels of leptin [54]. Leptin has been shown to directly increase proliferation of chick hepatocytes [55], although conversely administration of leptin was unable to restore replicative competence after partial hepatectomy [56], leaving conflicting evidence for the ability of leptin to promote hepatocellular proliferation. CXCL16 and Axl have both been shown to increase epithelial proliferation, yet we detected an increase in growth when hepatocytes were exposed to CXCL16. MCPs can induce proliferation, yet hepatocytes are known to not express the CCR2 and therefore hepatocytes should not directly respond to these chemokines. Hepatocytes do constituitively express CXCR2 and can respond to MIP-2 [57], [58] and possibly CXCL1. We found both CXCL1 and CXCL2 to be upregulated in conditioned media from CD11b isolated myeloid cells in steatotic livers and both of these cytokines were able to increase the growth of murine conditionally immortalized hepatocyte cells (HepT) and human HepG2 cells hepatocytes *in vitro*. Further, ELR-CXC chemokines have been shown to induce hepatocyte proliferation in culture [58]. We were further able to show that leptin was also capable of inducing growth of hepatocytes *in vitro*. Indirectly, increased activating chemokines in the steatotic liver may also influence resident stromal cells to impact the growth of hepatocytes.

In summary, this study demonstrates that there are significant changes in hepatocellular proliferation, influx of inflammatory cell populations and cytokine levels in the steatotic liver. The recognition of their roles in progression of NAFLD to end stage liver disease and a potential tumor-initiating role in the steatotic liver microenvironment may open the door for modulation of these cell populations and cytokines as part of novel therapies, especially for difficult-to-treat cancers such as HCC. Additional investigations are needed to understand the mechanisms by which these changes in inflammatory cell populations, cytokines, and the proliferation of hepatocytes have on the progression of NAFLD.

## Supporting Information

Table S1 **Inflammatory cell profiles in the spleen of normal vs steatotic mice.**
(DOCX)
