# Supplementary material for: Hepatocellular Proliferation Correlates with Inflammatory Cell and Cytokine Changes in a Murine Model of Nonalchoholic Fatty Liver Disease
Source: PLoS One. 2013 Sep 9;8(9):e73054. doi: 10.1371/journal.pone.0073054 (PMC3767686; doi:10.1371/journal.pone.0073054)
Supplement: Table S1 — Inflammatory cell profiles in the spleen of normal vs steatotic mice. (DOCX) [file pone.0073054.s001.docx]

**Table S1. Inflammatory cell profiles in the spleen of normal vs steatotic mice**

| **Cell Type** | **Normal** | **Steatotic** | **P Value** |
| --- | --- | --- | --- |
| CD3 | 27.58 | 36.275 | 0.0036 |
| CD3 CD4 | 51.02 | 50.975 | 0.9882 |
| CD3 CD8 | 37.16 | 39.05 | 0.4916 |
| CD3 CD25 | 10.592 | 14.45 | 0.0234 |
| CD3 CD62Ln | 8.036 | 10.5975 | 0.2968 |
| CD11b | 9.72 | 8.825 | 0.6062 |
| CD11b f480 | 16.52 | 12.74 | 0.1948 |
| CD11b Gr1hi | 23.84 | 14.9025 | 0.047 |
| CD11b Ly6cp Gr1p | 26.225 | 15.925 | 0.0496 |
| cd11b Ly6cn Gr1p | 22.18 | 24.275 | 0.5718 |
| cd19 | 66.2 | 64.625 | 0.6423 |
| cd11cp cd19n | 1.54 | 1.8025 | 0.0868 |
| NK | 2.498 | 2.7525 | 0.7138 |
